# The Effects of Virtual Reality-Based Task-Oriented Movement on Upper Extremity Function in Healthy Individuals: A Crossover Study

**DOI:** 10.3390/medicina61040668

**Published:** 2025-04-04

**Authors:** Tuba Maden, Halil İbrahim Ergen, Zarife Pancar, Antonio Buglione, Johnny Padulo, Gian Mario Migliaccio, Luca Russo

**Affiliations:** 1 Department of Physiotherapy and Rehabilitation, Faculty of Health Sciences, Gaziantep University, 27050 Gaziantep, Turkey; tuba.kmaden@gmail.com (T.M.); halilibrahimergen@yahoo.com (H.İ.E.); 2 Department of Physical Education and Sport, Faculty Sport Science, Gaziantep University, 27310 Gaziantep, Turkey; 3 Department of Theoretical and Applied Sciences, eCampus University, 22060 Novedrate, Italy; antonio.buglione@uniecampus.it (A.B.); luca.russo2@uniecampus.it (L.R.); 4 Department of Biomedical Sciences for Health, Università degli Studi di Milano, 20133 Milan, Italy; johnny.padulo@unimi.it; 5 Department of Human Sciences and Promotion of the Quality of Life, San Raffaele Rome Open University, 00166 Rome, Italy; 6 Athlete Physiology, Psychology and Nutrition Unit, Maxima Performa, 20126 Milan, Italy

**Keywords:** virtual reality, upper extremity, reaction time, joint position sense, task-oriented

## Abstract

*Background and Objectives*: Although virtual reality (VR) has been shown to be effective in rehabilitation through motor learning principles, its impact on upper extremity function, particularly in the context of console use, remains unclear. *Materials and Methods*: This study aimed to investigate the effects of VR-based task-oriented movement on the upper extremity of healthy individuals. A total of 26 healthy individuals performed task-oriented movements in both real and virtual environments in a randomized order. All participants completed a single session of task-oriented movements using a VR Goggle system in a virtual setting. Physiotherapists designed immersive VR-based experiences and 3D screen-based exergames for this study. Upper extremity function was assessed using several measures: joint position sense (JPS) of the wrist and shoulder was evaluated using a universal goniometer, reaction time was measured via a mobile application, and gross manual dexterity was assessed using the box-and-block test (BBT). Evaluations were conducted before and after the interventions. *Results*: The results showed that JPS remained similar between conditions, while BBT performance improved in both groups. However, the reaction time increased significantly only after VR intervention (*p* < 0.05). No significant period or carryover effects were observed across the parameters. These findings suggest that VR-based task-oriented training positively influences reaction time and supports hand function. Moreover, VR systems that simulate joint position sense similar to real-world conditions may be beneficial for individuals with musculoskeletal motor deficits. *Conclusions*: These results highlight the potential for integrating VR technology into rehabilitation programs for patients with neurological or orthopedic impairments, providing a novel tool for enhancing upper extremity function and injury prevention strategies.

## 1. Introduction

The use of virtual reality (VR) devices as joint kinematics monitoring systems in rehabilitation is rapidly rising [1]. The user in VR is fully immersed in the virtual environment and can interact with virtual objects in simulated environments, using controllers [2]. Immersive VR increases patients’ adherence to the rehabilitation protocol. VR systems provide more enjoyment and fun in long-term rehabilitation and follow patients easily [2]. VR systems are used in different rehabilitation areas, as well as health and fitness areas [3]. Specifically, in rehabilitation, these systems are effective in enabling real-time feedback, tracking, recording performance, and performing motor learning. It is easier to maintain patient motivation in VR during many repetitions than in actual performance. The number of repetitions is important for motor learning in rehabilitation. Repetitive and task-oriented movements show beneficial effects on facilitative motor skills [4]. Task-oriented movement is a crucial component of therapeutic intervention in rehabilitation [5].

VR systems include active range of motion, coordination, attention, and cognition exercises in each and every session [4]. Especially for upper extremity rehabilitation, game developers have aimed to improve the gross and fine dexterity and hand coordination of the users with movements such as hand pronation–supination and finger flexion–extension for human health [6]. The intensity, repetition, and target of the intervention can be arranged in the virtual environment to gain functionality and increase skill [7]. Thus, the therapeutic effect of these systems is rapidly increasing. Upper extremity functions improve by inducing motor control and learning in patients [8]. In addition, it is known that an environment enriched by motor learning principles is important for acquiring skills, and this property can be achieved by VR. It has been shown that visual signals can alter kinesthesia in healthy individuals [9]. In an immersive virtual environment, the user combines various signals (e.g., visual, motor, tactile, and muscle-related) to carry out quality motor movements [10]. Visual perception is different from the real environment, and by detecting the avatar of the user’s hands, the user reveals motor performance. This situation may cause the perception of reality to change, namely illusion in games [11,12]. The acute alteration of one’s kinesthesia sense can directly alter their proprioceptive sense and indirectly change the person’s reaction time and gross manual dexterity.

It has been shown that proprioception, which is thought to play a key role in every step of rehabilitation, develops in patients using technological systems such as exoskeleton robotic devices [13]. Most studies determined a set of tasks improving sensorimotor function [14,15,16]. Movement in VR includes three-dimensional space and isolated movements mostly in the transverse plane for functionality [15,16]. Although the use of increasingly widespread technological games on sick individuals is increasing, there is no consensus on the acute effect of technological devices on the proprioceptive system in healthy individuals [17]. Factors such as uniorbimanual tasks, simultaneous or delayed feedback, the type of tasks, and the plane on which movements are made may explain the lack of consensus. Since there is selective movement in task-oriented movement, the effect can be observed more clearly. In addition, it has been stated that VR interventions applied to individuals with various problems are effective on reaction time (RT) [18,19,20]. The RT, defined as the latent period between the stimulus given from a stimulus source and the response to this stimulus, reflects the activity process of the central nervous system represented by motor preparation and motor program [21,22]. RT is very important for functional movement. Information on VR’s effects on RT for healthy participants is limited in the literature. In a study conducted with healthy participants, figures during sports were simulated. It was observed that there was a significant improvement in RT in the group among which the VR intervention took place [23]. However, it is not known how factors affecting performance, such as reaction time and joint position sense, are acutely affected by games developed for health.

The effects of VR on the upper extremities have been extensively studied in different diseases. The number of studies examining the same effect in healthy individuals is limited in the literature. Knowing how upper extremity functions are affected by console use in healthy individuals will help people to design health games for rehabilitation and understand the effect of technology on healthy individuals. Whether joint position sense, reaction time, and upper extremity skills change or not may lead to the use of VR systems in different areas. The originality of this study lies in its focus on systematically investigating the acute effects of virtual reality (VR)-based task-oriented movement training on healthy individuals—a topic which remains underexplored in the literature. While VR applications have been extensively studied in clinical populations, particularly in individuals with neurological or orthopedic impairments, limited attention has been given to their effects on sensorimotor responses in non-clinical, healthy populations. Studying healthy individuals enables researchers to isolate the pure neurophysiological effects of VR interventions without confounding pathological factors, thereby providing clearer insight into the fundamental mechanisms of motor control and sensory adaptation elicited by VR. Furthermore, as VR technologies are increasingly being adopted in non-clinical domains such as fitness, sports performance, and preventive health, it becomes essential to evaluate their impact on motor function and proprioception in healthy users. Thus, this study not only bridges a significant gap in the literature but also highlights the potential of VR as a performance-enhancing and injury-preventive tool beyond traditional rehabilitation settings. In light of all these considerations, this study aimed to investigate the effect of virtual reality applied to a designed task-oriented movement of the upper extremity of healthy individuals.

## 2. Materials and Methods

### 2.1. Experimental Design and Research Rationale

This study was designed as a randomized, crossover trial to investigate the effects of virtual reality-based task-oriented training on upper extremity function in healthy individuals. The study protocol was approved by the Gaziantep University Local Ethics Committee for Clinical Research (decision no: 2022/404), in accordance with the ethical principles outlined in the Declaration of Helsinki. Prior to participation, all individuals were informed in detail about the study’s objectives, methodology, potential risks, and benefits. Participants provided written informed consent before enrollment, ensuring their voluntary participation. Confidentiality and anonymity of participant data were strictly maintained throughout the study. Participants were randomly assigned to one of two intervention sequences using a computer-generated randomization process. The crossover design ensured that each participant underwent both real and virtual training conditions, minimizing inter-individual variability. Furthermore, this study was registered at ClinicalTrials.gov under the identifier NCT06836310 (registered on 8 February 2025) to ensure transparency and the reproducibility of the findings. The trial registration provides a publicly accessible record of the study’s objectives, methodology, and anticipated outcomes.

### 2.2. Participants

**Figure 1:**
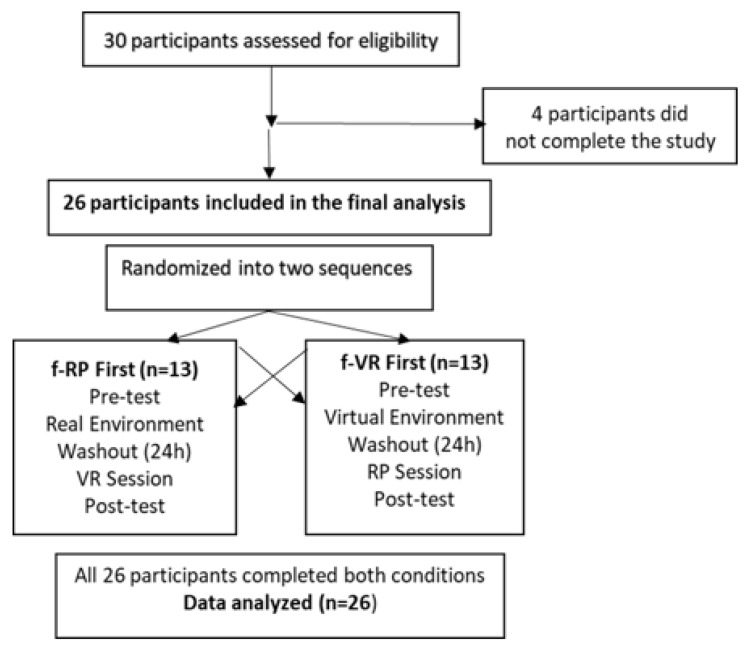
CONSORT flow diagram illustrating the crossover structure of the study. Participants were randomly assigned to either receive the real environment condition first (f-RP) or the virtual reality condition first (f-VR), with a 24 h washout period between sessions.

Twenty-six participants were included in this study. The sample size was determined through an a priori power analysis conducted using G*Power software (version 3.1). Assuming a medium effect size (Cohen’s d = 0.5), a significance level of α = 0.05, and a statistical power of 0.80, the analysis indicated that a minimum of 27 participants would be required for a within-subject design. This effect size was determined in reference to similar studies on short-term VR interventions in healthy adults and also supported by preliminary data collected during the early phase of the study, which served as an internal pilot. Initially, 30 healthy individuals were enrolled in the study. However, four participants were excluded due to their inability to complete both testing sessions. As a result, data from 26 participants were included in the final analysis, which was deemed acceptable considering the crossover design and the study’s exploratory nature. Demographic characteristics of the participants are presented in Table 1. The inclusion criteria were the following: (a) being over 18 years old, (b) volunteering to participate in the study, (c) having no upper extremity orthopedic disease (carpal tunnel syndrome, etc.). The exclusion criteria were (a) not having an upper extremity injury in the previous year, (b) the presence of rheumatic disease, (c) pregnancy, and (d) the presence of cervical disk herniation and thoracic outlet syndrome. The study was conducted and reported in accordance with the CONSORT 2010 guidelines for randomized crossover trials. A flow diagram outlining the participants’ progress throughout the study is provided in Figure 1.

### 2.3. Procedure and Randomization

After an initial screening, participants were randomly allocated into two groups: real performance first (f-RP) and virtual reality first (f-VR). Randomization was conducted using an online randomization tool (https://www.randomizer.org/ accessed on 25 January 2024) to ensure an unbiased assignment. The crossover design ensured that each participant experienced both conditions in a counterbalanced order, minimizing potential carryover effects. Participants in the f-RP group first performed the task-oriented movement in a real environment, followed by the same movement in a virtual reality setting. Participants in the f-VR group first completed the task-oriented movement in a virtual reality environment, followed by the same movement in a real setting. Each intervention session was conducted on two consecutive days to maintain consistency across conditions. Participants underwent a total of four assessments, administered before and after each intervention session.

To ensure the effectiveness of the crossover design, a clearly defined washout period was implemented. In this crossover study, a washout period of approximately 24 h (i.e., one day between sessions) was used between the two intervention conditions. This interval was selected based on the short-term nature of the VR-based intervention and previous research suggesting that acute motor and sensory effects of such applications tend to subside within this time frame. Moreover, statistical analysis revealed no significant carryover effects in any of the outcome variables (as shown in Table 2), supporting the adequacy of the chosen washout period. These assessments were conducted individually, in a controlled laboratory environment, and were supervised by experienced physiotherapists to ensure methodological consistency. This approach allowed for within-subject comparisons, reducing inter-individual variability and providing a robust evaluation of the intervention effects.

### 2.4. Intervention Protocol

**Figure 2:**
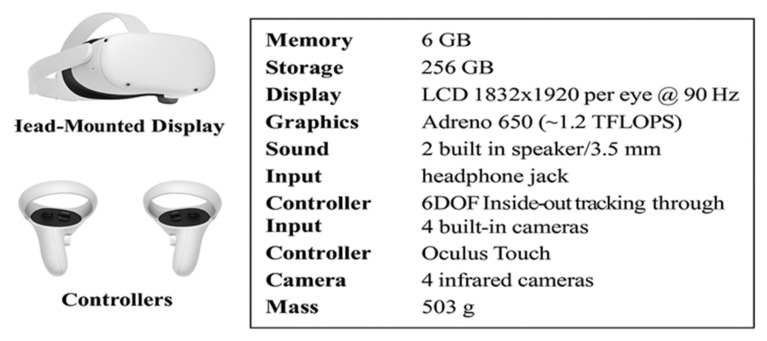
Technical features of the Oculus Quest 2 VR system.

A head-mounted display (HMD) designed for immersive virtual reality environments (Oculus Quest 2; Meta Quest, Menlo Park, CA, USA) was used in the VR sessions. An Oculus Quest 2 Head-Mounted Display and two Oculus Touch Controllers were employed in this study. In Figure 2, the main technical features of the Oculus Quest 2 VR system are reported. The virtual reality scenario was developed with Unity software (Unity Technologies, San Francisco, CA, USA, version 2017.1.1f1). The accessibility and interactivity were coded on the Visual Studio platform [1]. The virtual reality scenario and application were designed by the physiotherapist who had game developer training. All participants performed a single session of task-oriented movement with VR Goggle in a virtual environment. The intervention lasted 10 min, during which participants continuously performed the task-oriented ball-throwing activity. On average, each participant completed approximately 40 to 50 throws per session, depending on their self-paced rhythm. While the number of repetitions was not strictly controlled, all participants remained actively engaged throughout the entire intervention period. This estimated repetition range was considered sufficient to induce short-term neuromuscular activation. However, future studies are encouraged to standardize and document the number of repetitions to enhance control over training exposure. At the beginning, the physiotherapist taught the participant how to throw balls by guiding the movement, and then the participant was allowed to perform alone. The participants were asked to throw 3 balls into the pool in a virtual environment to demonstrate the intervention. The intervention allowed the participants to bilaterally use their upper extremities with repetitive task-oriented wrist movement.

**Figure 3:**
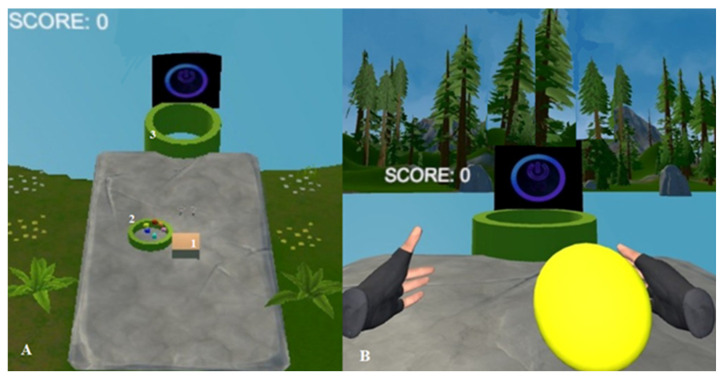
(**A**) General view, where A1 is the place of sitting, A2 is the ball pool, and A3 is the target pool, and (**B**) view of participant in the virtual environment before throwing.

The shoulder provides stability for producing task-oriented movement. In this study, the task-oriented movement comprised throwing the balls into the pool 2 m away. This pool was positioned linearly directly opposite the participant, with the head in a neutral position. It was the same for both virtual and real environments. When the balls entered the pool, the system provided visual and audible feedback (Figure 3). In addition, when the ball reached the pool, vibration was given by the console, providing tactile feedback to the participants. The physiotherapists tracked the participants in sync via Meta Quest on the mobile phone [9]. This study was designed as a preliminary investigation to evaluate the acute effects of a single session of VR-based task-oriented movement on upper extremity function, proprioception, and reaction time in healthy individuals. The purpose was to explore short-term neuromuscular responses and provide baseline data for future research. While one session may not be sufficient to produce long-term changes, it allowed researchers to isolate the immediate effects of the intervention without confounding factors such as adaptation, fatigue accumulation, or learning. Future studies should investigate the mid- and long-term impacts of repeated VR-based interventions with extended durations, in both clinical and non-clinical populations.

### 2.5. Outcome Measures

Joint position sense (JPS) was measured using a universal goniometer with a reproduction method. A 30 cm plastic goniometer (Baseline 12-1001; Fabrication Enterprises, Shenzhen, China) was used for the measurements. The measurements were performed using the evaluation positions recommended by the American Medical Association for goniometric measurements. To avoid the distraction of the participants, the measurements were taken in a quiet environment, and the participants were blindfolded to rule out visual clues.

The participants were asked to stay in an upright sitting position on a chair and have their feet resting on the floor with 90° hip and knee flexion, with their back unsupported. The participant’s arm was in the extended position during sitting. The shoulder of the participant was positioned with the goniometer at 90° flexion by a physiotherapist. The participant was asked to, in turn, return to the starting point (full extension) and then bring the shoulder to the half point of the movement. The axis of the goniometer was placed on the lateral aspect of the center of the humeral head, approximately 2.5 cm below the acromion process. The goniometer’s stationary arm was positioned parallel to the midaxillary line of the trunk and its moving arm parallel to the longitudinal axis of the humerus, pointing toward the lateral epicondyle. We determined the difference between the angular value that the participant should have achieved and the absolute angular error recorded [24,25]. All data for the outcome measures were collected using the dominant hand.

The shoulder was positioned in a neutral position, the elbow flexed to 90°, and the forearm in pronation for wrist assessment. The wrist of the participant was positioned to allow full movement and hang over the edge of the table for wrist JPS. The participant was asked to, in turn, bring the wrist to the end of the extension movement, then return to the starting point, and finally repeat the movement until reaching the half point of the range of motion by preventing the wrist from seeing the rest of the hand. The pivot point of the goniometer was on the lateral side of the os triquetrous, while the fixed arm was parallel to the os ulna, and the movements of the individual were followed with the movable arm of the goniometer with reference to the movable arm’s os metacarpal V. The difference between the angular value that the participant should have achieved and the absolute error score they reached was recorded in degrees by looking at the value on the goniometer [26]. The measurement was taken two times, and the average value was recorded by the same physiotherapist.

Reaction time was evaluated using the “Reaction Time Test” (RTT) mobile application developed by Brock Carpani, administered on an Apple iPhone 6S (MN122TU/A, 32 GB, iOS 9, 4.7 inch). Although this specific application has not undergone formal validation or reliability testing in peer-reviewed scientific studies, its structure aligns closely with conventional simple reaction time (SRT) paradigms. In this test, visual stimuli consisting of red, yellow, and green colors appeared sequentially on the screen, and participants were instructed to tap the screen as quickly as possible only when the green color appeared. A total of 25 trials were conducted. To standardize the testing conditions, the mobile device was placed in airplane mode, with internet turned off, screen brightness set to the maximum, and the sound turned off. Reaction times were recorded in milliseconds (ms). This task primarily assessed simple reaction time (SRT), as it required a rapid motor response to a single predetermined stimulus, despite the presence of visual distractors. Although multiple colors appeared, there was no response selection among different stimuli, which distinguished this test from a true choice reaction time task [27]. The test was performed with the participants’ dominant hand in a sitting position. The absence of a formal validation study for the “Reaction Time Test” is acknowledged as a limitation of the current study.

Gross manual dexterity was evaluated by the box-and-block Test (BBT). This test is widely used in both clinical and non-clinical populations, including healthy adults. It has been shown to have high test–retest reliability and construct validity for healthy individuals as well [28]. The test consists of moving, for one minute and one at a time, the maximum number of blocks from one side of a box to the other. The number of blocks is recorded as a score. The test is objective, easy to perform, valid, and reliable for healthy participants. The test was performed with the participant’s dominant hand in a sitting position. The measurement was taken two times, and the average value was recorded [28,29].

### 2.6. Statistical Analysis

The Statistical Package for the Social Sciences version 22.0 software (IBM Corp., Armonk, NY, USA) was used for descriptive statistical analysis. The Shapiro–Wilk test was used to examine the normality of the data. The data met the assumptions for the use of parametric analysis. The frequency in percent (%) and mean ± standard deviation (mean ± SD) of necessary variables were calculated for the descriptive analyses. The results were considered significant when *p* < 0.05. An independent-sample *t*-test was used to investigate the homogeneity between groups in terms of age, BMI, JPS, RTT, and BBT at baseline. Chi-square was used to investigate the homogeneity between groups with respect to gender and the dominant side. The paired-sample *t*-test was used in the comparison of the two measuring conditions, pre intervention and post intervention. The carryover effect and period effect were evaluated with NCSS 2020 (Statistical Software, 2020; NCSS, LLC, Kaysville, UT, USA). The period effect determined the importance of how individuals received treatments and compared the groups’ 4th evaluations. The carryout effect indicated whether the first treatment’s effect continued when starting the second treatment and was determined by comparing the 1st and 3rd evaluation of a group for long-term effects. The groups were combined, since it was seen that there were no carryover nor period effects in all the data. The change produced by a single intervention was calculated. To assess the presence of carryover and period effects in the crossover design, statistical analyses were performed prior to the main comparisons. The carryover effect was evaluated by comparing the sum of outcome scores from period 1 and period 2 between the two intervention sequences (f-VR first vs. f-RP first) using an independent-sample *t*-test. The period effect was assessed by comparing the differences in scores between the two periods across all participants, also using an independent-sample *t*-test. These analyses were conducted in accordance with standard procedures for two-treatment crossover trials. The results indicated no statistically significant carryover or period effects, confirming the validity of the crossover structure.

## 3. Results

**Figure 4:**
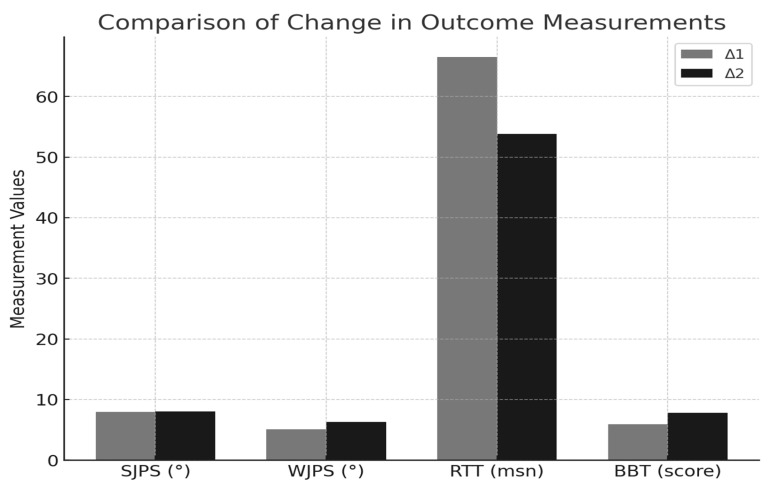
Comparison of the change in outcome measurements in all individuals. The bars represent the mean changes (Δ1 and Δ2) in shoulder joint position sense (SJPS), wrist joint position sense (WJPS), reaction time test (RTT), and box-and-block test (BBT) scores. Darker shades indicate Δ2 values.

The demographic characteristics of the participants were similar, as shown in Table 1. The outcome measures according to groups were homogeneous at baseline (*p* > 0.05). When the in-group comparisons were examined, only the RTT of the f-VR group was increased (*p* < 0.05) (Table 2). After both interventions, the joint position sense did not change, and the BBT values increased (Table 2). No period and carryover effects were observed in all parameters. When the changes made by the interventions in the parameters were compared, the results were similar (*p* > 0.05) (Table 3). The comparison of pre- and post-intervention changes in all individuals is presented in Table 4. As shown in Figure 4, the changes in outcome measurements (Δ1 and Δ2) are illustrated.

Although this study employed a crossover design in which all participants completed both intervention conditions, the demographic and baseline characteristics were presented separately according to the initial random assignment (f-RP and f-VR). This separation reflected the randomized order of condition exposure and was included to ensure transparency in reporting potential order-related influences. It did not imply independent group comparisons, as each participant served as their own control throughout the study.

The baseline differences observed in the wrist JPS and reaction time between the two conditions likely reflected the inherent variability in individual performance across different testing sessions rather than systematic randomization errors. Factors such as momentary fluctuations in attention, motor readiness, or circadian influences may have contributed to these variations. Despite these initial differences, the crossover design enabled within-subject comparisons, and no significant carryover or period effects were found. Thus, these baseline discrepancies were unlikely to bias the interpretation of the intervention effects.

## 4. Discussion

The present study aimed to investigate the effect of virtual reality applied to a task-oriented movement on upper extremity function, reaction time, and proprioception. In our study, although there was a significant improvement in the VR group for RTT, it was determined that the results were stable in the RP group. It was determined that there was a significant improvement in the upper extremity function for both groups. The improvement was similar in both groups’ wrist and shoulder joint position sense scores.

There are many factors affecting upper extremity functions. It has been stated that individuals with good hand function have better proprioception [25]. In our study, there was no improvement in proprioception for either group, but the groups’ shoulder and wrist joint position sense was similar. Task-oriented upper extremity movement designed in a virtual environment creates a shoulder and wrist position sensation similar to real performance. However, while the target movement is performed in the virtual environment, no movement occurs for the shoulder and wrist in the real world. Only the feeling of task-oriented movement of the upper extremity in the virtual world is perceived by individuals. In other words, when the target is achieved, there is a perception that the joints are moving like in the real environment. For this reason, task-oriented movement designed according to movement perception can be used in virtual environments in cases where movement is limited in the joint’s range of motion or when movement cannot be performed. Since task-oriented movement cannot be performed in the real environment in patients with motor component deficits, a real-like joint position sensation can be achieved with VR systems by creating a sense of movement. Our study on healthy individuals supports our view that the shoulder and wrist create a similar sense of joint position. However, the use of devices to increase sensory input is a developing topic in VR interventions and has not been fully clarified. In the VR environment, the sensory perception of reality with a system that provides only visual analysis cannot provide a sensory sensation like physical interaction with objects. Therefore, this situation cannot be used to achieve open interaction. The proprioceptive sensation formed via VR is very similar to that achieved in the real or physical world. For this reason, modern VR systems are often implicit systems, and control within them is exercised by surrogate methods, unlike the natural richness of object sensations [30]. So, although the perception of motion is similar, VR’s gains in joint position sense are not superior to the real environment because there is no explicit interaction. There was an improvement in upper extremity functions in this study; the lack of improvement in proprioception might have been related to the nature of VR systems.

Pourazar et al. showed that VR intervention was effective in influencing reaction time in children with cerebral palsy [19]. Petri et al. reported that VR applications caused positive changes in reaction time in athletes [23]. In another study, it was revealed that unrealistic computer games have the same structure as the virtual environment [31]. Drew and Waters reported that RTT and other perceptual–motor skills improved thanks to the video games they played with the participants [32]. Therefore, the results of our study support the literature. We think that a VR environment can lead to developments in RTT through its features. In addition, developing reaction time in VR can provide a new usage area in athlete and healthy individual evaluations.

It has been stated that evaluations made in a VR environment have advantageous aspects compared to traditional neurophysiological evaluations in the real environment. These are factors such as the participant’s unawareness of being evaluated, thus offering a more realistic performance, better participation, and greater generalization of learning [33]. We can say that applications made for therapy or intervention are similar in terms of these factors. In addition, increasing motor practice in a VR environment [31], the trainer’s ability to change environmental factors and give feedback in VR [34], and the individual’s protection against situations that may pose a danger outside of VR [34] are other factors that can affect evaluation in VR environments. That is, all these factors could explain the significant improvement seen in RTT.

It is known that the use of repetitive, focused, and real activities and actively applied interventions is important for supporting neuroplasticity [35]. For this purpose, traditional interventions have been complemented by virtual reality applications [36]. In fact, it has been stated that better results are obtained in upper extremity functions in VR applications compared to traditional rehabilitation applications of the same intensity [37]. There are promising studies on the effectiveness of VR-based therapy in improving upper extremity function in individuals who have had a stroke. However, these studies did not include a control group [38,39]. Although no improvement was detected in the f-VR group compared to the f-RE group for upper extremity functions in our study, the study design allowed for the effectiveness of VR to be observed clearly. While there was a significant improvement in the reaction time, there was no significant improvement in upper extremity function compared to the other group, which might have been due to the short intervention time.

Several previous studies have quantitatively demonstrated the benefits of virtual reality (VR) interventions in improving reaction time and upper extremity motor function. For instance, Witte et al. [40] integrated VR training into karate sessions and observed a significant reduction in response time for specific attack movements. The VR group’s response time to Gyaku-Zuki Jodan attacks in a virtual environment improved from 0.30 ± 0.05 s to 0.23 ± 0.06 s, while for Kizami-Zuki attacks it improved from 0.37 ± 0.05 s to 0.29 ± 0.05 s, with large effect sizes (η^2^ > 0.49). Similarly, Pumpho et al. [41] (2023) reported that a mobile VR application used in dual-task assessments among older adults showed strong concurrent validity for reaction time (r = 0.67) and TUG performance (r = 0.96), supporting the use of VR as a reliable tool in motor function evaluation. These findings illustrate the measurable benefits of VR-based training, particularly in enhancing neuromotor performance. Our current study builds upon this body of work by applying VR in healthy individuals to explore its short-term influence on reaction time and proprioceptive control.

The observed improvement in reaction time (RTT) could be attributed to the nature of the VR intervention, which included rapid visual and motor stimuli requiring immediate responses. Such stimuli are known to acutely engage the visual processing pathways, motor cortex, cerebellum, and sensorimotor integration circuits, thereby facilitating faster response execution. In contrast, joint position sense (JPS) is predominantly mediated by proprioceptive feedback mechanisms involving muscle spindles, joint receptors, and central integration over time. Unlike reaction time, improvements in proprioception typically require repeated and sustained exposure to movement patterns to induce sensorimotor adaptations. The single-session nature of the intervention might have been insufficient to elicit measurable changes in JPS. These differences highlight the specificity of neurophysiological adaptations in response to distinct sensorimotor demands.

This study had several limitations. First, the intervention period was short, consisting of a single session, which may be considered insufficient to observe meaningful improvements in proprioception or long-term changes in upper extremity function. However, the aim of the study was to evaluate the acute effects of a VR-based task-oriented movement intervention. Future clinical studies should explore longer intervention durations with repeated exposure to assess sustained adaptations. Second, while the virtual environment was designed to be rich in sensory stimuli, the use of a single task-oriented movement limited the diversity in sensorimotor challenges. According to motor learning principles, proprioceptive gains typically occur through repeated and variable joint movements across different ranges and positions. The use of only one standardized shoulder–hand position might have restricted the potential for proprioceptive adaptation. Future research should include multiple task designs targeting various upper extremity movement patterns and utilize game-based or interactive VR environments to better support sensorimotor development.

Our findings suggest that VR-based interventions may contribute to the rehabilitation of individuals with neurological disorders—such as stroke, multiple sclerosis, and cerebral palsy—by enhancing upper extremity function and motor control. Future studies should further investigate the clinical applicability of VR therapy across various rehabilitation settings and patient populations. Additionally, considering the critical role of proprioception and neuromuscular control in injury prevention, VR-based training may also serve as a valuable tool for athletes, particularly those involved in overhead sports, to improve motor coordination and reduce the risk of shoulder injuries. Future research should assess the long-term efficacy of VR training in sports injury prevention and performance enhancement.

## 5. Conclusions

This study demonstrated that virtual reality (VR)-based task-oriented training has a positive impact on reaction time and supports upper extremity function. The findings suggest that VR environments can simulate joint position sense similar to real-world conditions, making them a potential tool for individuals with musculoskeletal motor deficits. The ability of VR to provide interactive, engaging, and repetitive task-based exercises could enhance rehabilitation outcomes and injury prevention strategies. Despite these promising results, this study had certain limitations. First, the intervention duration was relatively short, which might have constrained the extent of improvements observed in upper extremity function and proprioception. Future studies with prolonged intervention periods are necessary to evaluate long-term effects. Second, while the VR environment was designed to be rich in sensory stimulation, additional enhancements incorporating haptic feedback and multisensory integration could further optimize motor learning and proprioceptive gains. Third, the study focused on a single task-oriented movement, limiting the generalizability of the results. Expanding future research to include diverse task-oriented exercises targeting multiple upper limb movements may provide a more comprehensive understanding of VR’s rehabilitative potential.

Future research should also explore the applicability of VR training in clinical settings for patients with neurological disorders, such as stroke, multiple sclerosis, and cerebral palsy. Additionally, given the role of proprioception and neuromuscular control in injury prevention, VR-based training may serve as an effective tool for athletes, particularly those engaged in overhead sports, to enhance motor coordination and reduce the risk of upper extremity injuries. Investigating the long-term impact of VR interventions on functional recovery, neuroplasticity, and athletic performance will be critical for the further integration of VR technology into rehabilitation and sports science.

## Figures and Tables

**Table 1:** Characteristics of individuals according to groups at baseline.

	f-RP	f-VR	t or X^2^	*p*
Age (years)	21.15 ± 1.06	24.46 ± 7.03	1.677	0.11 ^a^
Gender			0.000	1.000 ^b^
Female	7	7		
Male	6	6		
Dominant Side			2.167	0.480 ^b^
Right	11	13		
Left	2	-		
BMI (kg/m^2^)	22.42 ± 2.90	23.36 ± 3.92	0.698	0.492 ^a^
SJPS (°)	17.57 ± 4.46	14.00 ± 7.80	1.420	0.172 ^a^
WJPS (°)	3.15 ± 3.63	8.31 ± 8.45	2.020	0.060 ^a^
RTT (ms)	337.15 ± 54.07	407.46 ± 141.27	1.676	0.114 ^a^
BBT (score)	90.54 ± 11.49	91.15 ± 11.69	0.135	0.893 ^a^

^a^ Independent *t*-test; ^b^ Chi-squared test; BBT: box-and-block test score; RTT: reaction time; SJPS: shoulder joint position sense; and WJPS: wrist joint position sense.

**Table 2:** Pre-intervention and post-intervention values in the groups.

	f-RP			f-VR		
	Pre-Interv.	Post-Interv.	*p*	Pre-Interv.	Post-Interv.	*p*
SJPS (°)	17.57 ± 4.46	16.92 ± 6.14	0.918	14.00 ± 7.80	19.92 ± 6.13	0.070
WJPS (°)	3.15 ± 3.63	5.88 ± 6.54	0.176	8.31 ± 8.45	7.65 ± 4.34	0.660
RTT (ms)	337.15 ± 54.07	330.00 ± 58.19	0.088	407.46 ± 141.27	410.92 ± 168.82	0.000 *
BBT (score)	90.54 ± 11.49	92.38 ± 13.75	0.001 *	91.15 ± 11.69	95.23 ± 15.27	0.009 *

BBT: box-and-block test score; RTT: reaction time; SJPS: shoulder joint position sense; WJPS: wrist joint position sense; and * *p* < 0.05.

**Table 3:** Period effect (PEF) and carryover effect (COEF) analysis using independent-sample *t*-test.

Outcome	PEF (*p*)	COEF (*p*)
SJPS (°)	0.262	0.338
WJPS (°)	0.400	0.764
RTT (ms)	0.359	0.259
BBT (score)	0.281	0.419

**Table 4:** Comparison of the change in outcome measurements in all individuals.

	Δ1	Δ2	t	*p*
SJPS (°)	7.95 ± 5.63	8.05 ± 6.70	0.056	0.956
WJPS (°)	5.05 ± 3.43	6.27 ± 5.05	0.990	0.335
RTT (ms)	66.50 ± 83.24	53.85 ± 43.26	0.700	0.492
BBT (score)	5.90 ± 4.27	7.80 ± 7.24	1.198	0.246

BBT: box-and-block test score; RTT: reaction time; SJPS: shoulder joint position sense; and WJPS: wrist joint position sense.

## Data Availability

The data presented in this study are available upon request from the corresponding authors.

## References

[1] Carnevale A., Mannocchi I., Sassi M.S.H., Carli M., De Luca G., Longo U.G., Denaro V., Schena E. (2022). Virtual reality for shoulder rehabilitation: Accuracy evaluation of Oculus Quest 2. Sensors.

[2] Tao G., Garrett B., Taverner T., Cordingley E., Sun C. (2021). Immersive virtual reality health games: A narrative review of game design. J. Neuroeng. Rehabil..

[3] Greco G., Centrone C., Poli L., Silva A.F., Russo L., Cataldi S., Giustino V., Fischetti F. (2024). Impact of coastal walking outdoors and virtual reality indoor walking on heart rate, enjoyment levels, and mindfulness experiences in healthy adults. J. Funct. Morphol. Kinesiol..

[4] Lee H.S., Lim J.H., Jeon B.H., Song C.S. (2020). Non-immersive virtual reality rehabilitation applied to a task-oriented approach for stroke patients: A randomized controlled trial. Restor. Neurol. Neurosci..

[5] Esfahlani S.S., Thompson T., Parsa A.D., Brown I., Cirstea S. (2018). ReHabgame: A non-immersive virtual reality rehabilitation system with applications in neuroscience. Heliyon.

[6] Pillai A., Sunny M.S.H., Shahria M.T., Banik N., Rahman M.H.J.A.S. (2022). Gamification of upper limb rehabilitation in mixed-reality environment. Appl. Sci..

[7] Hegazy R.M., Alkhateeb A.M., Abdelmohsen A.M. (2022). Impact of virtual reality program on upper limb function post-stroke: A randomized controlled trial. Physiother. Q..

[8] Langhammer B., Sunnerhagen K.S., Lundgren-Nilsson Å., Sällström S., Becker F., Stanghelle J.K. (2017). Factors enhancing activities of daily living after stroke in specialized rehabilitation: An observational multicenter study within the Sunnaas International Network. Eur. J. Phys. Rehabil. Med..

[9] Dupraz L., Bourgin J., Giroux M., Barra J., Guerraz M.J. (2022). Involvement of visual signals in kinaesthesia: A virtual reality study. Neurosci. Lett..

[10] Proske U., Chen B. (2021). Two senses of human limb position: Methods of measurement and roles in proprioception. Exp. Brain Res..

[11] Kaneko F., Blanchard C., Lebar N., Nazarian B., Kavounoudias A., Romaiguère P. (2015). Brain regions associated with a kinesthetic illusion evoked by watching a video of one’s own moving hand. PLoS ONE.

[12] Kaneko F., Yasojima T., Kizuka T. (2007). Kinesthetic illusory feeling induced by a finger movement movie: Effects on corticomotor excitability. Neuroscience.

[13] Al-Whaibi R.M., Al-Jadid M.S., ElSerougy H.R., Badawy W. (2022). Effectiveness of virtual reality-based rehabilitation versus conventional therapy on upper limb motor function of chronic stroke patients: A systematic review and meta-analysis of randomized controlled trials. Physiother. Theory Pract..

[14] Aman J.E., Elangovan N., Yeh I.-L., Konczak J. (2015). The effectiveness of proprioceptive training for improving motor function: A systematic review. Front. Hum. Neurosci..

[15] De Santis D., Zenzeri J., Casadio M., Masia L., Riva A., Morasso P., Squeri V. (2015). Robot-assisted training of the kinesthetic sense: Enhancing proprioception after stroke. Front. Hum. Neurosci..

[16] De Santis D., Zenzeri J., Casadio M., Masia L., Morasso P., Squeri V. (2014). A new method for evaluating kinesthetic acuity during haptic interaction. Robotica.

[17] Valdés B.A., Khoshnam M., Neva J.L., Menon C. (2020). Robotics-assisted visual-motor training influences arm position sense in three-dimensional space. J. Neuroeng. Rehabil..

[18] Molhemi F., Monjezi S., Mehravar M., Shaterzadeh-Yazdi M.-J., Salehi R., Hesam S., Mohammadianinejad E. (2021). Effects of virtual reality vs conventional balance training on balance and falls in people with multiple sclerosis: A randomized controlled trial. Arch. Phys. Med. Rehabil..

[19] Pourazar M., Mirakhori F., Hemayattalab R., Bagherzadeh F. (2018). Use of virtual reality intervention to improve reaction time in children with cerebral palsy: A randomized controlled trial. Dev. Neurorehabil..

[20] Bisson E., Contant B., Sveistrup H., Lajoie Y. (2007). Functional balance and dual-task reaction times in older adults are improved by virtual reality and biofeedback training. Cyberpsychol. Behav..

[21] Botwinick J., Thompson L.W. (1966). Premotor and motor components of reaction time. J. Exp. Psychol..

[22] Weiss A.D. (1965). The locus of reaction time change with set, motivation, and age. J. Gerontol..

[23] Petri K., Emmermacher P., Danneberg M., Masik S., Eckardt F., Weichelt S., Bandow N., Witte K. (2019). Training using virtual reality improves response behavior in karate kumite. Sports Eng..

[24] Vafadar A.K., Côté J.N., Archambault P.S. (2016). Interrater and intrarater reliability and validity of three measurement methods for shoulder-position sense. J. Sport Rehabil..

[25] Ünlüer N.Ö., Ozkan T., Yaşa M.E., Ateş Y., Anlar Ö. (2019). An investigation of upper extremity function in patients with multiple sclerosis, and its relation with shoulder position sense and disability level. Somatosens. Mot. Res..

[26] Ergen H.İ., Keskinbıçkı M.V., Öksüz Ç. (2024). The effect of proprioceptive training on hand function and activity limitation after open carpal tunnel release surgery: A randomized controlled study. Arch. Phys. Med. Rehabil..

[27] Ioannou C.I., Hodde-Chriske F.L., Avraamides M.N., Altenmüller E. (2024). The impact of fine motor activities like playing musical instruments on the thickness and strength of the flexor digitorum muscle. J. Occup. Med. Toxicol..

[28] Mathiowetz V., Volland G., Kashman N., Weber K. (1985). Adult norms for the Box and Block Test of manual dexterity. Am. J. Occup. Ther..

[29] Tornero-Aguilera J.F., Jimenez-Morcillo J., Rubio-Zarapuz A., Clemente-Suárez V. (2022). Central and peripheral fatigue in physical exercise explained: A narrative review. Int. J. Environ. Res. Public Health.

[30] Zakharov A.V., Kolsanov A.V., Khivintseva E.V., Pyatin V.F., Yashkov A.V. (2021). Proprioception in Immersive Virtual Reality.

[31] Reid D.T. (2002). Benefits of a virtual play rehabilitation environment for children with cerebral palsy on perceptions of self-efficacy: A pilot study. Pediatr. Rehabil..

[32] Drew B., Waters J. (1986). Video games: Utilization of a novel strategy to improve perceptual motor skills and cognitive functioning in the non-institutionalized elderly. Cogn. Rehabil..

[33] Schultheis M.T., Rizzo A.A. (2001). The application of virtual reality technology in rehabilitation. Rehabil. Psychol..

[34] Holden M.K., Dyar T. (2002). Virtual environment training: A new tool for neurorehabilitation. J. Neurol. Phys. Ther..

[35] Liepert J., Bauder H., Miltner W.H., Taub E., Weiller C. (2000). Treatment-induced cortical reorganization after stroke in humans. Stroke.

[36] Shin J.-H., Kim M.-Y., Lee J.-Y., Jeon Y.-J., Kim S., Lee S., Seo B., Choi Y. (2016). Effects of virtual reality-based rehabilitation on distal upper extremity function and health-related quality of life: A single-blinded, randomized controlled trial. J. Neuroeng. Rehabil..

[37] Laver K.E., Lange B., George S., Deutsch J.E., Saposnik G., Crotty M. (2017). Virtual reality for stroke rehabilitation. Cochrane Database Syst. Rev..

[38] Merians A.S., Fluet G.G., Qiu Q., Saleh S., Lafond I., Davidow A., Adamovich S.V. (2011). Robotically facilitated virtual rehabilitation of arm transport integrated with finger movement in persons with hemiparesis. J. Neuroeng. Rehabil..

[39] Tsoupikova D., Stoykov N.S., Corrigan M., Thielbar K., Vick R., Li Y., Triandafilou K., Preuss F., Kamper D. (2015). Virtual immersion for post-stroke hand rehabilitation therapy. Ann. Biomed. Eng..

[40] Witte K., Droste M., Ritter Y., Emmermacher P., Masik S., Bürger D., Petri K. (2022). Sports training in virtual reality to improve response behavior in karate kumite with transfer to real world. Front. Virtual Real..

[41] Pumpho A., Kaewsanmung S., Keawduangdee P., Suwannarat P., Boonsinsukh R. (2023). Development of a mobile application for assessing reaction time in walking and TUG duration: Concurrent validity in female older adults. Front. Med..

